# On the use of GBLUP and its extension for GWAS with additive and epistatic effects

**DOI:** 10.1093/g3journal/jkab122

**Published:** 2021-04-19

**Authors:** Jie Zhang, Fang Liu, Jochen C Reif, Yong Jiang

**Affiliations:** Department of Breeding Research, Leibniz Institute of Plant Genetics and Crop Plant Research (IPK) Gatersleben, 06466 Stadt Seeland, Germany

**Keywords:** GWAS, GBLUP, Q +, K linear mixed model, epistatic effect

## Abstract

Genomic best linear unbiased prediction (GBLUP) is the most widely used model for genome-wide predictions. Interestingly, it is also possible to perform genome-wide association studies (GWAS) based on GBLUP. Although the estimated marker effects in GBLUP are shrunken and the conventional test based on such effects has low power, it was observed that a modified test statistic can be produced and the result of test was identical to a standard GWAS model. Later, a mathematical proof was given for the special case that there is no fixed covariate in GBLUP. Since then, the new approach has been called “GWAS by GBLUP”. Nevertheless, covariates such as environmental and subpopulation effects are very common in GBLUP. Thus, it is necessary to confirm the equivalence in the general case. Recently, the concept was generalized to GWAS for epistatic effects and the new approach was termed rapid epistatic mixed-model association analysis (REMMA) because it greatly improved the computational efficiency. However, the relationship between REMMA and the standard GWAS model has not been investigated. In this study, we first provided a general mathematical proof of the equivalence between “GWAS by GBLUP” and the standard GWAS model for additive effects. Then, we compared REMMA with the standard GWAS model for epistatic effects by a theoretical investigation and by empirical data analyses. We hypothesized that the similarity of the two models is influenced by the relative contribution of additive and epistatic effects to the phenotypic variance, which was verified by empirical and simulation studies.

## Introduction

Genome-wide association study (GWAS) and genome-wide prediction (GWP) are two extensively applied tools in the study of complex traits in human, animal, and plant populations (De Los Campos *et al.* 2010; Wray *et al.* 2013; Hickey *et al.* 2017; Evans *et al.* 2018). GWAS is used to dissect the genetic architecture and identify potential causal variants for the trait, whereas GWP exploits all genetic variants such as single nucleotide polymorphisms (SNPs) to predict the genetic values of unphenotyped individuals. Despite the different focuses, the statistical models utilized for GWAS and GWP share many common features.

The state-of-the-art model for GWAS in structured populations is the Q + K linear mixed model (Kennedy *et al.* 1992; Yu *et al.* 2006). In this model, the population structure is controlled by setting subpopulation effects as fixed covaraites (Q) and the cryptic relatedness is taken into account by a random term with a kinship matrix defining the genetic covariance between individuals (K). The marker effect under test is modeled as a fixed parameter and its significance is assessed by the likelihood ratio test (Lippert *et al.* 2011) or the *F*-test (Kang *et al.* 2008). The model has to be fitted once for each marker. Thus, the computational load can be very high for large-scale data sets as the estimation of variance components usually involves an iteration procedure. A widely applied efficient approximation approach is the P3D (population parameters previously determined) method (Zhang *et al.* 2010). Namely, the variance components are estimated only once in a “null model” without including any marker effect and then they are fixed throughout the test for all markers. It has become a standard GWAS approach implemented in many software packages such as EMMAX (Kang *et al.* 2010), GAPIT (Zhang *et al.* 2010), and rrBLUP (Endelman 2011).

For GWP, the most commonly applied model is the genomic best linear unbiased prediction (GBLUP; VanRaden 2008), which is also a linear mixed model. In this model, the genetic values for unphenotyped individuals are predicted via a genomic relationship matrix connecting the phenotyped and unphenotyped individuals. The genomic relationship matrix can also be used as the covariance matrix controlling the cryptic relatedness for GWAS. Then, the GBLUP model is exactly the “null model” of the Q + K model for GWAS. On the other hand, GBLUP is equivalent to the ridge regression best linear unbiased prediction (RR-BLUP; Whittaker *et al.* 2000; Meuwissen *et al.* 2001), in which all marker effects are explicitly modeled as random variables. With these observations, we can treat GBLUP as a bridge connecting the Q + K model for GWAS and the RR-BLUP model for GWP. Thus, an interesting question is whether GWAS can be directly performed using the GBLUP model. More precisely, whether a test statistic can be constructed directly using the GBLUP model in which the marker effects were modeled as random.

In fact, it has been proposed to perform GWAS directly based on the GBLUP model using the estimated effects (Wang *et al.* 2012) or the proportion of phenotypic variance explained by the markers (Dikmen *et al.* 2013; Wang *et al.* 2014). However, test statistics were not constructed in these approaches. A more meaningful approach is to construct a test statistic based on the estimated marker effects in GBLUP. This has also been considered in previous studies in the framework of ridge regression (Malo *et al.* 2008; Shen *et al.* 2013). Namely, a test statistic following t-distribution can be formed by taking the estimated marker effect divided by the square root of its prediction error variance (PVE), or equivalently, its posterior variance from the Bayesian perspective (Chen *et al.* 2017). Nevertheless, when the number of markers is large, the estimated marker effects in the GBLUP model and the corresponding test statistics are usually over shrunken, which leads to low detection power (Wang *et al.* 2020b).

Interestingly, it was demonstrated that an alternative test statistic can be constructed using the estimated marker effect in GBLUP divided by the square root of its variance in the sense of Henderson (1975) instead of the PVE, and it was observed that the *P*-values produced in this method were almost identical to those obtained in the Q + K GWAS model (Duarte *et al.* 2014). Later, it was mathematically proved that the new test statistic is identical to the P3D approximated test statistic in the Q + K model (Bernal Rubio *et al.* 2016). Although the proof was only for the special case that no fixed covariates are included in the GBLUP model, it has been accepted by the community that the two GWAS approaches are equivalent (Chen *et al.* 2017; Lu *et al.* 2018; Aguilar *et al.* 2019) and the new approach was termed “GWAS by GBLUP” (Legarra *et al.* 2018). It was also generalized to window-based or SNP-set association tests (Chen *et al.* 2017). Nevertheless, it is very common to include fixed covariates (*e.g.*, an intercept, subpopulation, environmental effects, and known QTL effects) in the GBLUP model. Thus, a strict mathematical proof for the general case is needed to confirm the equivalence between “GWAS by GBLUP” and the standard GWAS approach.

Recently, the concept of “GWAS by GBLUP” has been extended to the 2D scan for epistatic effects (Ning *et al.* 2018; Wang *et al.* 2020a). That is, the GBLUP model can be extended to add a second random term with an epistatic genomic relationship matrix as covariance matrix, called EGBLUP (Jiang and Reif 2015; Martini *et al.* 2016). Similar to the equivalence between GBLUP and RR-BLUP, the EGBLUP model is equivalent to a model explicitly including the additive effects of all markers and the epistatic effects for all pairs of markers. Because of this equivalence, the test statistics for the epistatic effects for all pairs of markers can be efficiently calculated by fitting the EGBLUP model only once. This approach was termed rapid epistatic mixed-model association analysis (REMMA; Ning *et al.* 2018). On the other hand, the standard Q + K model for GWAS can also be extended to test the significance of marker epistatic effects (Lippert *et al.* 2013; Xu 2013). Thus, it is necessary to investigate the relationship between REMMA and the extended Q + K method. Similar to the case of additive effects, one might expect that they are equivalent because of the connections between the underlying models. However, there have not been any theoretical or empirical studies comparing the two approaches.

In this study, we aimed to answer two questions: (1) Is “GWAS by GBLUP” equivalent to the standard GWAS approach for marker additive effects in the general case (when fixed covariates are presented in the model)? (2) Is REMMA equivalent to the extended standard GWAS approach for marker epistatic effects? For the first question, we gave an affirmative answer by providing a strict mathematical proof that “GWAS by GBLUP” is equivalent to the P3D approximated Q + K approach. Moreover, the equivalence is valid not only for single SNP-based test but also for window-based test in which the additive effects of a group of SNPs are tested together. For the second question, we first made a theoretical comparison, which indicated that the two approaches are not equivalent in general. Then we verified our theoretical finding with empirical data analysis. Based on the empirical results, we hypothesized that the different performance of the two approaches is influenced by the ratio of the additive to the epistatic variance component and verified the hypothesis with a simulation study.

## Materials and methods

### GWAS by the Q + K linear mixed model

The Q + K linear mixed model (Yu *et al.* 2006) has the following form:

(1) $$y=X\beta +m_{i}a_{i}+g+e.$$

The notations and assumptions are the following: y is the *n*-dimensional vector of phenotypic records. β is the *k*-dimensional vector of covariate effects. X is the corresponding *n *×* k* design matrix. *a_i_* is the additive effect of the *i*-th marker and *m_i_* is the *n*-dimensional vector of marker codings. g denotes the *n*-dimensional vector of polygenic background effects and e is the residual term. In the model, β and *a_i_* are assumed to be fixed parameters and $g∼N(0,K\sigma_{g}^{2})$, where K is a matrix of kinship coefficients estimated by the pedigree or genomic data. $e∼N(0,I\sigma_{e}^{2})$ and Cov(e,g)=0.

Note that in model (1), we assumed that each genotype has only one record and the same order of individuals was applied to g and y. Therefore, the design matrix for g is an identity matrix and hence omitted. This assumption is made throughout the manuscript in order to simplify the presentation of our theoretical results. The general case is treated in the Supplementary Notes (Supplementary File S1).

In GWAS, we are mainly interested in the significance of the marker effect *a_i_*. It can be assessed using the following test statistic, which follows a *t*-distribution with n−k−1 degrees of freedom:

(2) $$z_{i,Q+K}=\frac{{\hat{a}}_{i,f}}{\sqrt{\text{Var}({\hat{a}}_{i,f})}},$$

where ${\hat{a}}_{i,f}$ is the best linear unbiased estimate of *a_i_* and a subscript “f” is added to emphasize that the marker effect is modeled as a fixed parameter.

### GWAS by the GBLUP model

The GBLUP model is of the following form:

(3) y=Xβ+g+e,

where the notations are the same as in (1) except that $g∼N(0,G\sigma_{g}^{2})$, where G is a genomic relationship matrix derived from marker information. If we take K=G in (1), the only difference between (1) and (3) is that there is no marker effect term in (3). Thus, the GBLUP model can be treated as the “null model” of the Q + K GWAS model.

It is well-known that GBLUP is equivalent to the following RR-BLUP model (Habier *et al.* 2007):

(4) y=Xβ+Ma+e,

where a is the *p*-dimensional vector of additive effects for all markers, $a∼N(0,I\sigma_{a}^{2})$ and M is an *n *×* p* matrix of marker profiles. In particular, the genomic relationship matrix usually takes the form G=MM′/c, where *c* is a scaling factor (*e.g.*, VanRaden 2008). Then the equivalence between (3) and (4) can be seen by taking g=Ma and then we have $\sigma_{a}^{2}=\sigma_{g}^{2}/c$. Without loss of generality, we assume *c *=* *1 from now on and this will simplify the presentation of our results and their proofs. The assumption is released in the Supplementary Notes (Supplementary File S1).

Because of the equivalence, the estimate $\hat{a}$ in (4) as well as $\text{Var}(\hat{a})$ can be efficiently calculated via linear transformations of the estimate $\hat{g}$ and $\text{Var}(\hat{g})$ from model (3). Namely,

$$\begin{array}{c} \hat{a}=M\prime G^{-1}\hat{g},\\ \text{Var}(\hat{a})=M\prime G^{-1}\text{Var}(\hat{g})G\prime^{-1}M. \end{array}$$

Note that here Var(·) denote the variance of BLUP in the sense of Henderson (1975).

Then, GWAS can be performed using test statistics constructed by $\hat{a}$ and $\text{Var}(\hat{a})$ (Duarte *et al.* 2014). The form of the test statistic is similar to (2) and follows a t-distribution with n−k−1 degrees of freedom:

(5) $$z_{i,\mathrm{GBLUP}}=\frac{{\hat{a}}_{i,r}}{\sqrt{\text{Var}({\hat{a}}_{i,r})}},$$

where ${\hat{a}}_{i,r}$ is the *i*-th entry of the vector $\hat{a}$, *i.e.*, the estimated effect of the *i*-th marker. The subscript “r” was added to emphasize that the marker effect was modeled as a random variable. This is the so-called “GWAS by GBLUP” approach (Legarra *et al.* 2018).

### GWAS for epistasis by the extended Q + K model

The extension of the Q + K model (1) for testing epistatic effects between markers is straightforward (Reif *et al.* 2011; Lippert *et al.* 2013). The model can be described as follows:

(6) $$y=X\beta +m_{i}a_{i}+m_{j}a_{j}+(m_{i}\circ m_{j})aa_{ij}+g+e,$$

where *a_i_* and *a_j_* are the additive effects of the *i*-th and the *j*-th markers (i≠j), $m_{i}$ and $m_{j}$ are the corresponding marker coding vectors, *aa_ij_* is the epistatic effect between the two markers, $m_{i}\circ m_{j}$ is the element-wise product of the two vectors $m_{i}$ and $m_{j}$. In the model, *a_i_*, *a_j_*, and *aa_ij_* are assumed to be fixed parameters. All other notations and assumptions are the same as in (1).

Since the genomic relationship matrix G=MM′ only takes the additive marker effects into account, it may be necessary to control the epistatic background effects in addition to the additive background effects when testing the epistatic effect between markers. With this purpose, the model (6) can be modified to include one more random term with an epistatic genomic relationship matrix (Xu 2013; Jiang *et al.* 2017; Runcie and Crawford 2019):

(7) $$y=X\beta +m_{i}a_{i}+m_{j}a_{j}+(m_{i}\circ m_{j})aa_{ij}+g_{A}+g_{AA}+e,$$

where $g_{A}$ and $g_{AA}$ are the vectors of additive and additive-by-additive genetic values, respectively, and all other notations are the same as in (6). In this model, we assume that $g_{A}∼N(0,G\sigma_{A}^{2})$ and $g_{AA}∼N(0,H\sigma_{AA}^{2})$, where H is the epistatic genomic relationship matrix calculated as follows (Jiang and Reif 2020):

(8) $$H=\frac{1}{2}(G\circ G-(M\circ M)(M\circ M)`).$$

As there are two genomic relationship matrices in model (7), we call it “Q + 2K” for convenience.

In both models, the significance of the epistatic effect *aa_ij_* can be assessed by a Wald test (Xu 2013), in which the test statistic has the form

$$w_{ij,Q+2K}=\frac{{\hat{aa}}_{ij,f}^{2}}{\text{Var}({\hat{aa}}_{ij,f})},$$

which is the square of the z-score statistic similar to (2) and follows asymptotically the $\chi^{2}$ distribution with one degree of freedom. The subscript “f” again indicates that the epistatic effect is modeled as a fixed variable.

### GWAS for epistasis by the EGBLUP model (REMMA)

The extended genomic best linear unbiased prediction (EGBLUP) model is an natural extension of the classic GBLUP model to include epistasis (Jiang and Reif 2015; Martini *et al.* 2016). It has the following form:

(9) $$y=X\beta +g_{A}+g_{AA}+e,$$

where all notations are the same as in (7).

Similar to the equivalence between GBLUP and RR-BLUP, the EGBLUP model was proved to be equivalent to the following model, which explicitly includes the epistatic effects between all pairs of markers (Jiang and Reif 2015):

(10) y=Xβ+Ma+Qaa+e,

where all notations are the same as in (4), except that aa is the vector of epistatic effects for all pair of markers, $\mathrm{aa}∼N(0,I\sigma_{aa}^{2}),\;Q$ is an n×p(p−1)/2 dimensional matrix whose columns are products of two distinct columns in M, *i.e.* $m_{i}\circ m_{j}$ for any *i*, *j* such that 1≤i<j≤p. For the equivalence between (9) and (10), we just need to take $g_{A}=\mathrm{Ma},\;g_{AA}=\mathrm{Qaa}$ and note that H=QQ′ (Jiang and Reif 2015). Then we have $\sigma_{A}^{2}=\sigma_{a}^{2},\;\sigma_{AA}^{2}=\sigma_{aa}^{2}$.

REMMA (Ning *et al.* 2018) is a rapid GWAS algorithm for epistatic effects based on EGBLUP. Its rationale is similar to “GWAS by GBLUP” for additive effects, *i.e.*, the estimates $\hat{\mathrm{aa}}$ and $\text{Var}(\hat{\mathrm{aa}})$ in (10) can be obtained by linear transformations of ${\hat{g}}_{AA}$ and $\text{Var}({\hat{g}}_{AA})$ in (9):

$$\begin{array}{c} \hat{\mathrm{aa}}=Q\prime H^{-1}{\hat{g}}_{AA}\\ \text{Var}(\hat{\mathrm{aa}})=Q\prime H^{-1}\text{Var}({\hat{g}}_{AA})H\prime^{-1}Q. \end{array}$$

Thus, one only needs to fit the model (9), which is computationally much more efficient than (10).

Then the hypothesis $H_{0}:aa_{ij}=0$ for any pair of markers *i* and *j* can be tested using the Wald statistic:

$$w_{ij,\mathrm{REMMA}}=\frac{{\hat{aa}}_{ij,r}^{2}}{\text{Var}({\hat{aa}}_{ij,r})}.$$

Here the subscript “r” is again to emphasize that in this approach the epistatic effect is modeled as a random variable.

### Comparison of GWAS models

For GWAS with additive effects, we compared the standard Q + K approach (1) with the “GWAS by GBLUP” approach (3). Only a theoretical comparison was made as it was enough to show the equivalence. For GWAS with epistatic effects, we compared the performances of the following three approaches: Q + K (6), Q + 2K (7), and REMMA (9). These models were first compared by theory and then by analyzing empirical and simulated data. The Q + K model was implemented by the FastLMM software package (Lippert *et al.* 2011). The Q + 2K model was implemented by ourselves using the statistical software R (R Core Team 2019) and the package BGLR (Pérez and de Los Campos 2014). The REMMA approach was implemented in Ning *et al.* (2018).

### Data sets

Four published data sets were used in this study: (1) A maize data set consisting of 2815 inbred accessions preserved mostly at the National Plant Germplasm System in the United States (Romay *et al.* 2013). The collection was genotyped by the genotyping-by-sequencing technology, which produced 681,257 SNP markers. The trait we analyzed is the growing degree days from planting to the day that 50% of the plants show silk. (2) A rice diversity panel consisted of 413 inbred accessions collected from 82 countries (Zhao *et al.* 2011). The panel was genotyped by an Affymetrix single SNP array and there were in total 44,100 SNP markers. Phenotypic data of plant height were analyzed in this study. (3) A wheat data set comprised of 1604 single-cross hybrids from a factorial design of 15 male and 120 female parental lines (Zhao *et al.* 2015). The parental lines were genotyped by an Illumina Infinium assay resulting of 17,372 high-quality SNPs. The trait under consideration was grain yield. (4) A mouse data set that contained 1304 genotypes from the *F*_10_ generation of an intercross line, each genotyped with 1470 SNPs (Jarvis and Cheverud 2011). The analyzed phenotype was reproductive fat pad weight.

As the purpose of empirical data analyses was to compare the resulting test statistics obtained in different models instead of investigating the full structure of epistatic interactions across the genome, it is not necessary to perform GWAS for epistatic effects with all pairs of markers in each data set. In order to reduce the computational load, we applied a pruning procedure to the markers based on linkage disequilibrium (LD) with a threshold of $r^{2}<0.2$, a window size of 1 Mb and a step size of 10 kb. That is, pairs of markers in the initial window with *r*^2^ values above 0.2 were noted, and the markers were greedily pruned until no such pairs remained. Then the window moved with a step size of 10 kb and the procedure was repeated. The LD pruning was done using the software PLINK (Purcell *et al.* 2007). The final number of markers as well as other information of each data set was summarized in Table 1.

**Table 1:** Summary information of the data sets

Data set	Species	Trait	Number of genotypes	Number of markers	Reference
1	Maize	Days to silking	2,279	1,690	Romay *et al.* (2013)
2	Rice	Plant height	383	1,732	Zhao *et al.* (2011)
3	Wheat	Grain yield	1,604	3,497	Zhao *et al.* (2015)
4	Mouse	Fat pad weight	1,304	1,407	Jarvis and Cheverud (2011)

### Simulation study

To further compare the performance of the three GWAS models for epistatic effects (Q + K, Q + 2K, and REMMA), we performed a simulation study. The purpose was to test the hypothesis that the behavior of these models was affected by the relative contribution of the additive and epistatic genetic effects to the phenotypic variance in the data set. For the motivation of the hypothesis, we refer to the *Results*.

The simulation was based on the genotypic data of the rice data set. The following formula was used to generate the simulated phenotypes:

$$y=g_{A}+g_{AA}+e,$$

where $g_{A}∼N(0,G\sigma_{A}^{2}),\;g_{AA}∼N(0,H\sigma_{AA}^{2})$, and $e∼N(0,I\sigma_{e}^{2})$. Using all markers, we calculated the genomic relationship matrices G following VanRaden (2008) and H using (8). We considered seven levels of $\sigma_{A}^{2}/\sigma_{AA}^{2}$ ratios (0.25,0.5,1,2,4,8,16) and nine levels of broad-sense heritability $h^{2}=(\sigma_{A}^{2}+\sigma_{AA}^{2})/(\sigma_{A}^{2}+\sigma_{AA}^{2}+\sigma_{e}^{2})$ (from 0.1 to 0.9 with a step of 0.1). For each of the 63 combinations, the simulation was repeated five times.

Note that we did not simulate any additive or epistatic QTL effects, for which there were two reasons. First, our main purpose was to compare the test statistics (and the resulting *P*-values) of different models for the epistatic effects of all marker pairs instead of investigating the QTL detection power. Secondly, if we simulated QTL effects, the test statistics for the QTLs would be more accurately estimated in the model which we chose to perform the simulation. This could potentially generate biased results.

Before comparing the GWAS models using the simulated data sets, we estimated the additive and epistatic variance components for all simulated data sets using model (9) with the restricted maximal likelihood method, which was obtained in REMMA. We found that although the estimated ratio ${\hat{\sigma }}_{A}^{2}/{\hat{\sigma }}_{AA}^{2}$ was significantly correlated with the simulated ratio across data sets (*P *<* *0.001), the correlation was only moderate (*r *=* *0.56). This result was not unexpected as the additive and epistatic covariance matrices were significantly correlated (*P *<* *0.001) due to the LD among markers. In fact, the correlation between G and H was 0.684 (maize), 0.681 (rice), 0.688 (wheat), and 0.437 (mouse). Thus, the contributions of additive and epistatic effects were mixed and it was difficult for the model to rediscover the simulated variance components.

In view of the above results, for each simulated $\sigma_{A}^{2}/\sigma_{AA}^{2}$ and *h*^2^ value, we defined an interval containing the simulated value as a criterion to filter the simulated data sets (Supplementary Tables S1 and S2). We only kept the data sets in which the estimated ${\hat{\sigma }}_{A}^{2}/{\hat{\sigma }}_{AA}^{2}$ and ${\hat{h}}^{2}$ values fell into the corresponding intervals. Further simulations were performed until for each of the 63 combinations of $\sigma_{A}^{2}/\sigma_{AA}^{2}$ and *h*^2^ values, there were five simulated data sets that fulfilled the criterion. Thus, in total, 315 data sets were used to compare the performance of the three GWAS models for epistatic effects.

For each simulated data set, we performed GWAS of the additive-by-additive epistatic effects for all pairs of markers using the three models. The test statistics and $-{\;\text{log}\;}_{10}(p)$ values were recorded. Pairwise comparison was made between REMMA and Q + 2K, as well as between Q + K and Q + 2K. In each simulated data set, we calculated the correlation between the $-{\;\text{log}\;}_{10}(p)$ values obtained in the two models being compared. As the data sets were simulated with a wide range of heritabilities, we classified them into three classes: ${\hat{h}}^{2}\geq 0.7,\;0.4\leq {\hat{h}}^{2}<0.7$, and ${\hat{h}}^{2}<0.4$. Note that within each class, the data sets had different ${\hat{\sigma }}_{A}^{2}/{\hat{\sigma }}_{AA}^{2}$ ratios. Thus, for each class of data sets, we obtained a number of data points, each representing the correlation between the $-{\;\text{log}\;}_{10}(p)$ values of the two models in a specific data set. Then, these data points were plotted against the ${\;\text{log}\;}_{2}({\hat{\sigma }}_{A}^{2}/{\hat{\sigma }}_{AA}^{2})$ values. In this way, we studied the influence of the ${\hat{\sigma }}_{A}^{2}/{\hat{\sigma }}_{AA}^{2}$ ratio on the performance of different GWAS models.

### Data availability

All empirical data sets used in this study have been published in previous studies. Supplementary File S1 contains the generalized mathematical proofs of the results in this study. The R code implementing the P3D approximated Q + 2K GWAS model for epistatic effects is provided in Supplementary File S2. The R code used to generate the simulated data is provided in Supplementary File S3. A sample phenotypic and genotypic data, which is a subset of the rice data, are provided in Supplementary Files S4 and S5, respectively. Supplementary material is available at figshare: https://doi.org/10.25387/g3.14356598.

## Results

### The equivalence between GWAS by GBLUP and by the Q + K model for additive effects

In this section, we compare the “GWAS by GBLUP” approach (3) and the standard Q + K approach (1). More precisely, we compare the corresponding test statistics (5) and (2). Note that in the case of no fixed covariates (*i.e.*, without the term Xβ in the models), it has been proved that the two test statistics are the same (Bernal Rubio *et al.* 2016). Thus, our aim is to investigate the general case.

Before we start, we need to make the following assumption: The variance components $\sigma_{g}^{2}$ and $\sigma_{e}^{2}$ of the Q + K model (1) are not re-estimated for each marker. Instead, they are estimated only once from the “null model”, which is the GBLUP model (3), and are then fixed throughout the test for all markers. This is the so-called P3D approximation of the Q + K GWAS approach mentioned in the *Introduction*. It is very important to consider the P3D approximation instead of the precise approach because it ensures that the estimated variance components are the same for the two test statistics. The same assumption was needed in the proof of Bernal Rubio *et al.* (2016).

Using Henderson’s mixed model equations (Henderson 1975), we know that the best linear unbiased estimate of *a_i_* and its variance in model (1) are the following:

(11) $$\begin{array}{cc} {\hat{a}}_{i,f} & =\frac{m_{i}^{\prime }\mathrm{Ty}}{m_{i}^{\prime }Tm_{i}},\\ \text{Var}({\hat{a}}_{i,f}) & =\frac{\sigma_{e}^{2}}{m_{i}^{\prime }Tm_{i}}, \end{array}$$

where $T=V^{-1}-V^{-1}X{\left(X\prime V^{-1}X\right)}^{-1}X\prime V^{-1}$, V=I+λG, and $\lambda =\sigma_{g}^{2}/\sigma_{e}^{2}$.

Replacing ${\hat{a}}_{i,f}$ and $\text{Var}({\hat{a}}_{i,f})$ in (2) by (11), we see that the test statistic for the *i*-th marker in model (1) is:

(12) $$z_{i,Q+K}=\frac{m_{i}^{\prime }\mathrm{Ty}}{\sigma_{e}\sqrt{m_{i}^{\prime }Tm_{i}}}.$$

On the other hand, the best linear unbiased prediction of a and its variance from model (3) is:

(13) $$\begin{array}{cc} \hat{a} & =\frac{\sigma_{a}^{2}}{\sigma_{e}^{2}}M\prime \mathrm{Ty},\\ \text{Var}(\hat{a}) & =\sigma_{a}^{2}I-{\left(\frac{\sigma_{e}^{2}}{\sigma_{a}^{2}}I+M\prime \mathrm{SM}\right)}^{-1}\sigma_{e}^{2}, \end{array}$$

where $S=I-X{\left(X\prime X\right)}^{-1}X$.

Taking the *i*-th component in (13), we obtain the best linear unbiased prediction for *a_i_*:

(14) $${\hat{a}}_{i,r}=\frac{\sigma_{a}^{2}}{\sigma_{e}^{2}}m_{i}^{\prime }\mathrm{Ty}.$$

However, a simplified formula for the variance of ${\hat{a}}_{i,r}$ is not straightforward from (13). Here we just write:

(15) $$\text{Var}({\hat{a}}_{i,r})=\text{Var}{\left(\hat{a}\right)}_{i,i},$$

where $\text{Var}{\left(\hat{a}\right)}_{i,i}$ denotes the *i*-th diagonal element of the matrix $\text{Var}(\hat{a})$.

Replacing ${\hat{a}}_{i,r}$ and $\text{Var}({\hat{a}}_{i,r})$ in (5) by (14), we see that the test statistic for the *i*-th marker in model (3) is:

(16) $$z_{i,\mathrm{GBLUP}}=\frac{\sigma_{a}^{2}m_{i}^{\prime }\mathrm{Ty}}{\sigma_{e}^{2}\sqrt{\text{Var}{\left(\hat{a}\right)}_{i,i}}}.$$

The key result in this section is the following:

(17) $$\text{Var}{\left(\hat{a}\right)}_{i,i}=\frac{\sigma_{a}^{4}}{\sigma_{e}^{2}}m_{i}^{\prime }Tm_{i}.$$

Using (17) and comparing (12) and (16), we can see that

(18) $$z_{i,\mathrm{GBLUP}}=z_{i,Q+K},\;\;\;\text{for}\;\text{any}\;i.$$

Thus, we have proved that the test statistics of the GBLUP model are equal to the P3D approximated test statistics from the Q + K model. This justifies the rationale of GWAS by GBLUP. The mathematical details for the derivation of (11), (13), and (17) are provided in Appendix.

In addition, the equivalence also holds for window-based test in which the additive effects of a group of SNPs are tested together (Chen *et al.* 2017). Namely, we consider the following model:

$$y=X\beta +Wa_{w}+g+e,$$

where $a_{w}$ is the vector of additive effects of *s* markers in the window being tested, W is the corresponding *n *×* s* matrix of marker profiles, and all other notations are the same as in (1). The null hypothesis is *H*_0_: $a_{w}=0$. The proof in such a general case is provided in the Supplementary Notes (Supplementary File S1).

### The theoretical difference between GWAS by REMMA, Q + K, and Q + 2K for epistatic effects

In this subsection, we make a theoretical comparison of GWAS for epistatic effects by REMMA (9), the extended Q + K (6), and the Q + 2K model (7). Note that the extended Q + K model only controlled the additive polygenic effects by the random term g, while the Q + 2K model and REMMA controlled both additive and epistatic polygenic effects through two random terms $g_{A}$ and $g_{AA}$. Thus, the Q + K model is not expected to be equivalent to REMMA or the Q + 2K model. In the remaining part of this subsection, we focus on the comparison of REMMA and Q + 2K.

We observe that (9) is the “null model” of (7) in the sense that (9) is obtained by removing the terms $m_{i}a_{i},\;m_{j}a_{j}$ and $(m_{i}{}^{\circ}m_{j})aa_{ij}$ in (7). This relationship is very similar to the case of GWAS for additive effects, *i.e.*, (3) is the “null model” of (1). Since we have proved that GWAS by GBLUP is equivalent to GWAS by Q + K for additive effects, one may expect that the same holds true for GWAS by REMMA and by Q + 2K for epistatic effects, *i.e.*

(19) $$w_{ij,\mathrm{REMMA}}=w_{ij,Q+2K},\;\;\;\text{for}\;\text{any}\;i\;\text{and}\;j.$$

However, a further investigation of the two models does not support the above hypothesis. To clarify this point, we introduce the following auxiliary model:

(20) $$y=X\beta +(m_{i}{}^{\circ}m_{j})aa_{ij}+g_{A}+g_{AA}+e,$$

in which all notations and assumptions are the same as in (7). Let ${\tilde{aa}}_{ij}$ be the best linear unbiased estimate of *aa_ij_* in the above model. We can also construct a test statistic:

$${\tilde{w}}_{ij}=\frac{{\tilde{aa}}_{ij}}{\text{Var}({\tilde{aa}}_{ij})}.$$

Let $g_{T}=g_{A}+g_{AA}$ and $V=G\sigma_{A}^{2}/\sigma_{AA}^{2}+H$. We can rewrite model (20) in the following form:

(21) $$y=X\beta +(m_{i}{}^{\circ}m_{j})aa_{ij}+g_{T}+e,$$

where $g_{T}∼N(0,V\sigma_{AA}^{2})$. With these notations, the model (9) can be written as:

(22) $$y=X\beta +g_{T}+e.$$

If we treat $m_{i}{}^{\circ}m_{j}$ as the coding vector of a new “marker,” the epistatic effect *aa_ij_* then becomes the “main effect” of this marker. With this point of view, the relationship between (21) and (22) is exactly the same as that between (1) and (3). Therefore, using the same argumentation line as in the proof of (18), we have the following result:

(23) $$w_{ij,\mathrm{REMMA}}={\tilde{w}}_{ij}.$$

Thus, comparing the two test statistics $w_{ij,Q+2K}$ with $w_{ij,\mathrm{REMMA}}$ is equivalent to comparing $w_{ij,Q+2K}$ with ${\tilde{w}}_{ij}$. The latter is much easier as the epistatic effect *aa_ij_* was treated as fixed parameters in both models. And the difference between models (7) and (20) is clear. Namely, the additive effects of the two markers (*a_i_* and *a_j_*) whose interaction effect is the target of test are included in (7), but not in (20). Based on this observation, we can anticipate that the two statistics $w_{ij,\mathrm{MKLMM}}$ and ${\tilde{w}}_{ij}$ are not likely to be equal, *i.e.* (19) may not be true in general.

### Comparing GWAS by REMMA, Q + K, and Q + 2K for epistatic effects with empirical data

**Figure 1:**
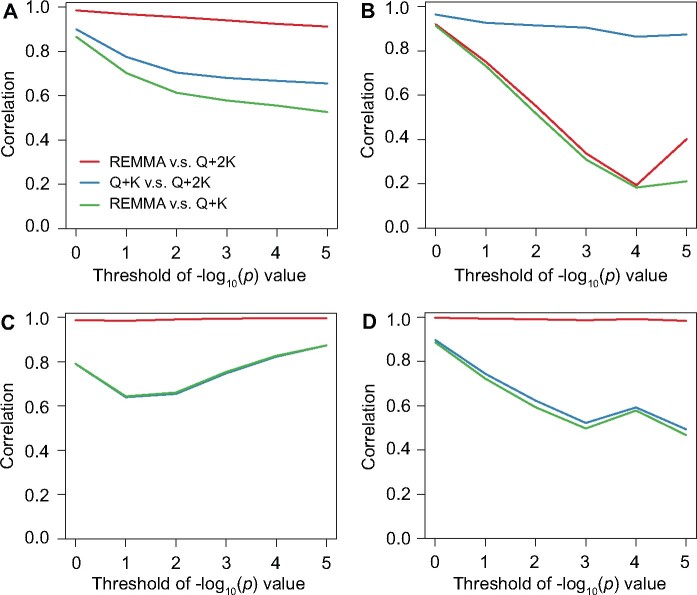
The correlation of $-{\;\text{log}\;}_{10}(p)$ values of different GWAS approaches (REMMA, Q + K, and Q + 2K) for epistatic effects in the (A) maize, (B) rice, (C) wheat, and (D) mouse data set. Correlations were calculated for the marker pairs whose $-{\;\text{log}\;}_{10}(p)$ values of epistatic effects were above a threshold *d* in at least one approach. Different values of *d* (from 0 to 5 with a step of 1) were considered.

As the theoretical investigation in the last subsection indicated that the three GWAS approaches (REMMA, Q + K, and Q + 2K) are not equivalent, we compared their performances with four empirical data sets (see *Materials and Methods*) in this subsection. Results were presented in Figure 1.

When all pairs of markers were considered, we observed that the correlation between the $-{\;\text{log}\;}_{10}(p)$ values obtained in REMMA and Q + 2K was high in all four data sets (Supplementary Figure S1), which seems contradicting to the theoretical results. However, when a certain threshold *d* of $-{\;\text{log}\;}_{10}(p)$ value was used to filter the marker pairs [*i.e.*, we only considered those marker pairs whose $-{\;\text{log}\;}_{10}(p)$ values were above *d* in at least one of the two models], the correlation sharply decreased to 0.19 as the threshold increased to *d *=* *4 in the rice data set. Although the correlation increased to 0.40 when *d *=* *5, it is still low. Thus, it is possible that the epistatic effect of a pair of markers is significant in one model but not in the other. In contrast, the correlation only slightly decreased as the increase of the threshold in the maize data set, and it was not affected by the threshold in the wheat and the mouse data set.

For the comparison of the Q + 2K and Q + K model, we observed that the correlation of the $-{\;\text{log}\;}_{10}(p)$ values decreased as the increase of the threshold in three of the four data sets. But in this case, it was the mouse data set in which we observed the largest amount of decrease of the correlation (*r *=* *0.49 when *d *=* *5). In the maize data set, the correlation also decreased to 0.66. In contrast, the correlation remained high (above 0.87) in the rice data set. These results clearly verified our theoretical findings that the test statistics obtained in the three models are not the same in general. For each of the four data sets, the $-{\;\text{log}\;}_{10}(p)$ values of all pairs of markers with $-{\;\text{log}\;}_{10}(p)>3$ in at least one of the three models were listed in Supplementary Table S3.

Based on the theoretical investigations in the last subsection, we can already infer the factors affecting the performance of different models. We already knew that the difference between REMMA [or equivalently, the auxiliary model (20)] and Q + 2K (7) is that REMMA does not include the additive effects of the two markers as fixed covariates when testing their epistatic effects, which seems unreasonable at a first glance, as the influence of the additive effects should be considered in the assessment of the epistatic effect. In fact, REMMA does take the influence of additive effects into account, but not as fixed covariates. Rather, the additive effects of all markers are implicitly included in the random polygenic term $g_{A}$. In view of this, we could anticipate that when the additive effects make a larger contribution to the total phenotypic variance than the epistatic effects, the Q + 2K model would have a stronger control of the additive effects than REMMA. On the other hand, the difference between the Q + K (6) and the Q + 2K model is that in Q + 2K there is an additional random polygenic term $g_{AA}$, which implicitly models the epistatic effects of all pairs of markers. Therefore, if the relative contribution of additive effects is much larger than the epistatic effects, the extra term $g_{AA}$ in Q + 2K would become unimportant and the performance of Q + 2K and Q + K should be similar. As the contribution of the additive effects relative to the epistatic effects can be measured by the ratio of their variance components $\sigma_{A}^{2}/\sigma_{AA}^{2}$, we can make the following hypothesis: As the increase of the $\sigma_{A}^{2}/\sigma_{AA}^{2}$ ratio, the similarity between the REMMA and the Q + 2K model decreases and that between the Q + 2K and the Q + K model increases.

With the above hypothesis, we estimated the ratio $\sigma_{A}^{2}/\sigma_{AA}^{2}$ in each data set (Table 2). The results provided a first evidence supporting our hypothesis: The mouse data set had the smallest ratio $\sigma_{A}^{2}/\sigma_{AA}^{2}$ and we observed that REMMA and Q + 2K nearly had the same performance, while the difference between Q + 2K and Q + K was large (Figure 1). The ratio $\sigma_{A}^{2}/\sigma_{AA}^{2}$ for the rice data set was the largest and in this case, REMMA differed greatly from Q + 2K, while the Q + 2K and Q + K performed similarly (Figure 1).

**Table 2:** The estimated genomic heritability and ratio of additive to epistatic variance components in the four data sets

Parameter	Data set			
	Maize	Rice	Wheat	Mouse
$h_{G}^{2}$	0.837	0.746	0.804	0.631
$\sigma_{A}^{2}/\sigma_{AA}^{2}$	2.499	9.394	3.907	0.823

### A simulation study on the influence of $\sigma_{A}^{2}/\sigma_{AA}^{2}$

**Figure 2:**
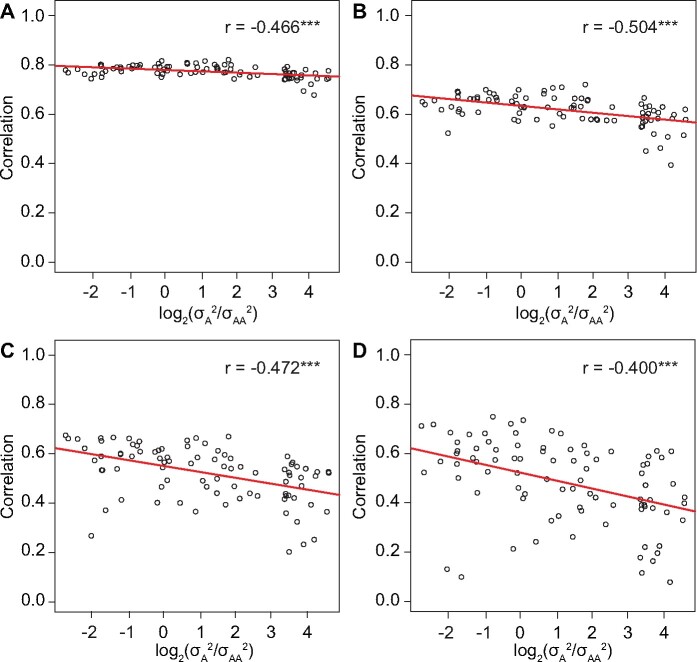
The correlations between the $-{\;\text{log}\;}_{10}(p)$ values for the epistatic effects obtained using the REMMA and the Q + 2K model in simulated data sets with $h^{2}\geq 0.7$ and different $\sigma_{A}^{2}/\sigma_{AA}^{2}$ ratios. Each point in the figure represented the correlation between the $-{\;\text{log}\;}_{10}(p)$ values from the two models calculated in a specific simulated data set. The overall correlation between the correlations and the ${\;\text{log}\;}_{2}(\sigma_{A}^{2}/\sigma_{AA}^{2})$ values across all data sets was displayed as the *r* value together with an indication of significance (*P < 0.1, **P < 0.05, ***P < 0.01). A threshold of $-{\;\text{log}\;}_{10}(p)$ values was applied to filter the marker pairs. Namely, only the marker pairs whose $-{\;\text{log}\;}_{10}(p)$ values were above the threshold in at least one of the two models were considered. In different panels, distinct threshold values were applied: (A) 1, (B) 2, (C) 3, and (D) 4.

To further verify our hypothesis, we compared the performance of the three GWAS approaches for epistatic effects with simulated data (see *Materials and Methods*). First, we focused on the comparison between REMMA and the Q + 2K model. When the heritability is above 0.7, we observed that the correlation between the $-{\;\text{log}\;}_{10}(p)$ values obtained in the two models was negatively correlated with the ${\;\text{log}\;}_{2}(\sigma_{A}^{2}/\sigma_{AA}^{2})$ value (Figure 2). The absolute value of the overall correlation was moderate (between 0.4 and 0.5) and significant (*P* < 0.01). As in the empirical data analysis, we applied a threshold of $-{\;\text{log}\;}_{10}(p)$ values to filter the marker pairs included in the analysis. The negative correlation was observed in all cases with four different thresholds. We also investigated the cases where the heritability is between 0.4 and 0.7 or below 0.4. In both cases, the correlation between the $-{\;\text{log}\;}_{10}(p)$ values obtained in the two models was also negatively correlated with the ${\;\text{log}\;}_{2}(\sigma_{A}^{2}/\sigma_{AA}^{2})$ value, but the absolute value of the overall correlation was lower than the case with heritability above 0.7 (Supplementary Figures S2 ad S3). These results clearly indicated that the similarity between REMMA and the Q + 2K model decreases as the increase of the $\sigma_{A}^{2}/\sigma_{AA}^{2}$ ratio, which supported our hypothesis.

**Figure 3:**
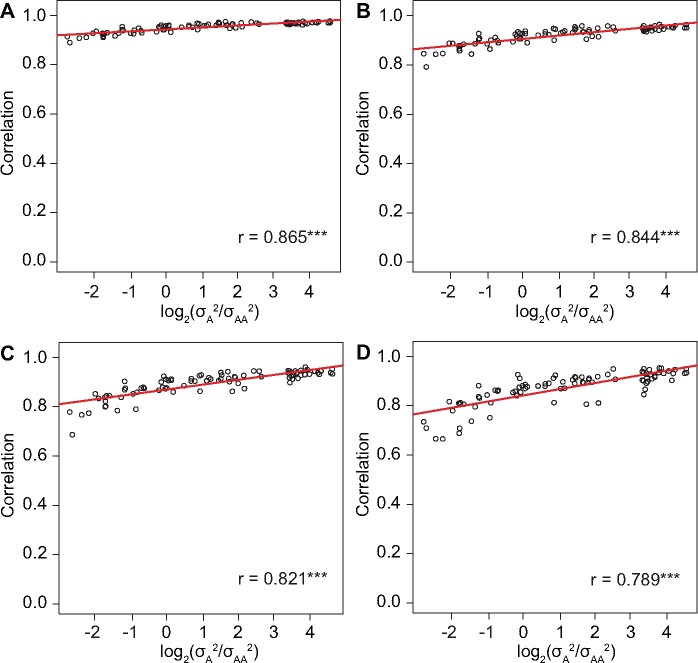
The correlations between the $-{\;\text{log}\;}_{10}(p)$ values for the epistatic effects obtained using the Q + K and the Q + 2K model in simulated data sets with $h^{2}\geq 0.7$ and different $\sigma_{A}^{2}/\sigma_{AA}^{2}$ ratios. Each point in the figure represented the correlation between the $-{\;\text{log}\;}_{10}(p)$ values from the two models calculated in a specific simulated data set. The overall correlation between the correlations and the ${\;\text{log}\;}_{2}(\sigma_{A}^{2}/\sigma_{AA}^{2})$ values across all data sets was displayed as the *r* value together with an indication of significance (*P < 0.1, **P < 0.05, ***P < 0.01). A threshold of $-{\;\text{log}\;}_{10}(p)$ values was applied to filter the marker pairs. Namely, only the marker pairs whose $-{\;\text{log}\;}_{10}(p)$ values were above the threshold in at least one of the two models were considered. In different panels, distinct threshold values were applied: (A) 1, (B) 2, (C) 3, and (D) 4.

Then, we compared the performances of the Q + K and the Q + 2K model in the simulated data sets. We observed that the correlation between the $-{\;\text{log}\;}_{10}(p)$ values obtained in the two models was positively correlated with the ${\;\text{log}\;}_{2}(\sigma_{A}^{2}/\sigma_{AA}^{2})$ value in the case with heritability above 0.7 (Figure 3). The overall correlation was high and significant. The same trend was observed in the cases with lower heritabilities, although the overall correlations were lower (Supplementary Figures S4 and S5). Moreover, the trend was not affected by the applied threshold to filter the markers. Thus, the results indicated that the similarity between the Q + K and Q + 2K model increases as the increase of the $\sigma_{A}^{2}/\sigma_{AA}^{2}$ ratio, which supported the second part of our hypothesis.

## Discussion

The goal of GWAS is to identify specific genomic regions harboring loci with relatively large effects while controlling the polygenic genetic background, which is partially contributed by those genomic regions we want to identify. The polygenic background effects can be modeled using GBLUP, a GWP model partitioning the observed phenotypic variation across the genome into the polygenic effect contributed by all markers and the residual. Thus, GWAS can be performed using the estimated marker effects in the GBLUP model, which was termed “GWAS by GBLUP” (Legarra *et al.* 2018). In this study, we provided a general proof of the equivalence between “GWAS by GBLUP” and the P3D approximated standard Q + K GWAS approach for additive effects. Thus, there is no distinction in the computational efficiency of the two methods. However, the situation of GWAS for epistatic effects is different. On the one hand, we showed that REMMA is not equivalent to Q + 2K, which is a natural extension of the standard Q + K method for additive effects. On the other hand, it was reported that REMMA is much faster than the extension of Q + K to epistatic effects (Ning *et al.* 2018). Thus, REMMA is computationally more efficient than Q + 2K, because the computational load of the extended Q + K and Q + 2K is similar if the P3D approximation is applied. Therefore, we may wish to profit from the efficiency of REMMA and meanwhile, not to lose information from the Q + 2K approach. According to our empirical and simulation study, the *P*-values generated by REMMA are more similar to those obtained in the Q + 2K approach when the ratio of additive to epistatic variance component $\sigma_{A}^{2}/\sigma_{AA}^{2}$ is not large. In view of this result, we may suggest to check the estimated ratio in the data set before deciding the strategy of GWAS for epistatic effects. If the ratio is not too high, we can rely on the REMMA method and benefit from its fast speed. Otherwise, it may be beneficial to run both models and compare the results.

Then, it is natural to ask the question which threshold of the $\sigma_{A}^{2}/\sigma_{AA}^{2}$ ratio should be applied. From our results with empirical data, it seems that the threshold could be at least 4, because the estimated ratio in the wheat data set was 3.9 (Table 1) and the performances of REMMA and Q + 2K were quite similar and independent of the applied threshold of $-{\;\text{log}\;}_{10}(p)$ values to filter the markers (Figure 1C). However, it was not supported by the results with simulated data. Even in the case where the $\sigma_{A}^{2}/\sigma_{AA}^{2}$ ratio is 1, the correlation between the $-{\;\text{log}\;}_{10}(p)$ values of the two models became lower than 0.6 when a threshold of 3 was applied. Therefore, further studies are needed to clarify such inconsistency, maybe through analyzing more empirical data sets and/or performing more comprehensive simulations based on different genotypic data.

In this study, we only considered additive-by-additive epistasis when discussing GWAS approaches for epistatic effects. Recently, the REMMA approach has been generalized to take all three types of digenic epistatic effects into account with remarkable computational efficiency (Wang *et al.* 2020a). The algorithm was termed REMMAX and the underlying model can be treated as an extended RR-BLUP model including the additive, dominance, and digenic epistatic effects of all markers. On the other hand, the extension of the standard Q + K GWAS model for all types of digenic epistatic effects has been developed (Xu 2013). Theoretically, our argumentation line for the equivalence between the test statistics of REMMA (9) and those from the auxiliary model (20) also works for REMMAX. Thus, it would be very interesting to compare REMMAX with approaches extending the standard Q + K model using empirical and simulated data.

As a final remark, we emphasize that “GWAS by GBLUP” is not equivalent to the exact Q + K approach since it is equivalent to the P3D approximation. The genetic and residual variance used for calculating the test statistic are estimated in GBLUP, which is a null model excluding any marker fixed effects. But when a marker is tested in the exact Q + K GWAS model, the estimated genetic and residual variance will be different from those estimated in the null model. Hence, the test statistic resulted from the exact Q + K model will also be different. Although it was reported that the approximated test statistics were highly correlated with the exact ones (Zhang *et al.* 2010), they tended to be conservative and potentially resulted in lower power (Zhou and Stephens 2012). Nevertheless, the P3D approximated approach avoids the estimation of variance parameters marker-by-marker and is computationally more efficient, especially for large-scale data sets, as implemented in many new fast GWAS algorithms (Loh *et al.* 2015; Runcie and Crawford 2019; Jiang *et al.* 2019).

## Funding

J.Z. is supported by China Scholarship Council (CSC scholarship No.201906350045). The GeneBank2.0 project (J.C.R.) has received funding from the Federal Ministry of Education and Research of Germany (grant FKZ031B0184A).

## Conflicts of interest

None declared.

## Appendix

### Two results in linear algebra

We list the following two results in linear algebra, which are needed in the remaining subsections:

Lemma
**A.1**. *Let* $A_{m\times m},B_{m\times n},C_{n\times m},D_{n\times n}$*be four matrices. Suppose that* A*and* D*are invertible, then we have*

$$\begin{array}{c} {\left(\begin{array}{cc} A & B\\ C & D \end{array}\right)}^{-1}=\left(\begin{array}{cc} {\left(A-BD^{-1}C\right)}^{-1} & -A^{-1}B{\left(D-CA^{-1}B\right)}^{-1}\\ -{\left(D-CA^{-1}B\right)}^{-1}CA^{-1} & {\left(D-CA^{-1}B\right)}^{-1} \end{array}\right).\\ {\left(A-BD^{-1}C\right)}^{-1}=A^{-1}+A^{-1}B{\left(D-CA^{-1}B\right)}^{-1}CA^{-1}. \end{array}$$

Lemma
**A.2**. *If two matrices* $A_{n\times m}$*and* $L_{n\times k}$*satisfy* L′A=0*and* $\Omega_{n\times n}$*is positive-definite, then we have*

$$\Omega -\Omega L{\left(L\prime \Omega L\right)}^{-1}L\prime \Omega =A{\left(A\prime \Omega^{-1}A\right)}^{-1}A\prime .$$

Lemma A.1 can be found in many text books of linear algebra (*e.g.*, Bernstein 2009). The proof of Lemma A.2 can be found in Verbyla (1990).

### The proof of Equation (11)

The Q + K linear mixed model (1) can be rewritten as follows:

$$y=\tilde{X}\tilde{\beta }+g+e,$$

where $\tilde{X}=(X|m_{i}),\;\tilde{\beta }=(\beta \prime ,a_{i,f})\prime$.

From Henderson’s mixed model equations (Henderson 1975), we know that the best linear unbiased estimation of the fixed effects are the following:

(A1) $$\hat{\tilde{\beta }}={\left(\tilde{X}\prime V^{-1}\tilde{X}\right)}^{-1}\tilde{X}\prime V^{-1}y,\;\;\;\text{Var}(\hat{\tilde{\beta }})={\tilde{C}}_{11}\sigma_{e}^{2},$$

where $V=I+\lambda G,\;\lambda =\sigma_{g}^{2}/\sigma_{e}^{2}$ and ${\tilde{C}}_{11}$ is defined via the following equation:

$${\left(\begin{array}{cc} \tilde{X}\prime \tilde{X} & \tilde{X}\prime \\ \tilde{X} & I+\lambda^{-1}G^{-1} \end{array}\right)}^{-1}=\left(\begin{array}{cc} {\tilde{C}}_{11} & {\tilde{C}}_{12}\\ {\tilde{C}}_{12}^{\prime } & {\tilde{C}}_{22} \end{array}\right).$$

Using Lemma A.1, we can calculate that

(A2) $$\begin{array}{cc} {\tilde{C}}_{11} & ={\left(\tilde{X}\prime \tilde{X}-\tilde{X}\prime {\left(I+\lambda^{-1}G^{-1}\right)}^{-1}\tilde{X}\right)}^{-1}\\ & ={\left(\tilde{X}\prime (I-{\left(I+\lambda^{-1}G^{-1}\right)}^{-1})\tilde{X}\right)}^{-1}={\left(\tilde{X}\prime V^{-1}\tilde{X}\right)}^{-1}. \end{array}$$

Using (A2), we can rewrite (A1) as follows:

(A3) $$\begin{array}{cc} \left(\begin{array}{c} \hat{\beta }\\ {\hat{a}}_{i,f} \end{array}\right) & ={\left(\begin{array}{cc} X\prime V^{-1}X & X\prime V^{-1}m_{i}\\ m_{i}^{\prime }V^{-1}X & m_{i}^{\prime }V^{-1}m_{i} \end{array}\right)}^{-1}\left(\begin{array}{c} X\prime V^{-1}y\\ m_{i}^{\prime }V^{-1}y \end{array}\right)\\ \text{Var}\left(\begin{array}{c} \hat{\beta }\\ {\hat{a}}_{i,f} \end{array}\right) & ={\left(\begin{array}{cc} X\prime V^{-1}X & X\prime V^{-1}m_{i}\\ m_{i}^{\prime }V^{-1}X & m_{i}^{\prime }V^{-1}m_{i} \end{array}\right)}^{-1}\sigma_{e}^{2}. \end{array}$$

Using (A3) and Lemma A.1, we can derive the explicit expressions for ${\hat{a}}_{i,f}$ and $\text{Var}({\hat{a}}_{i,f})$ as follows:

$$\begin{array}{c} {\hat{a}}_{i,f}={\left(m_{i}^{\prime }V^{-1}m_{i}-m_{i}^{\prime }V^{-1}X{\left(X\prime V^{-1}X\right)}^{-1}X\prime V^{-1}m_{i}\right)}^{-1}m_{i}^{\prime }V^{-1}y\\ -{\left(m_{i}^{\prime }V^{-1}m_{i}-m_{i}^{\prime }V^{-1}X{\left(X\prime V^{-1}X\right)}^{-1}X\prime V^{-1}m_{i}\right)}^{-1}m_{i}^{\prime }V^{-1}X{\left(X\prime V^{-1}X\right)}^{-1}X\prime V^{-1}y\\ =\frac{m_{i}^{\prime }(V^{-1}-V^{-1}X{\left(X\prime V^{-1}X\right)}^{-1}X\prime V^{-1})y}{m_{i}^{\prime }(V^{-1}-V^{-1}X{\left(X\prime V^{-1}X\right)}^{-1}X\prime V^{-1})m_{i}}=\frac{m_{i}^{\prime }\mathrm{Ty}}{m_{i}^{\prime }Tm_{i}},\\ \text{Var}({\hat{a}}_{i,f})={\left(m_{i}^{\prime }V^{-1}m_{i}-m_{i}^{\prime }V^{-1}X{\left(X\prime V^{-1}X\right)}^{-1}X\prime V^{-1}m_{i}\right)}^{-1}\sigma_{e}^{2}\\ =\frac{\sigma_{e}^{2}}{m_{i}^{\prime }(V^{-1}-V^{-1}X{\left(X\prime V^{-1}X\right)}^{-1}X\prime V^{-1})m_{i}}=\frac{\sigma_{e}^{2}}{m_{i}^{\prime }Tm_{i}}, \end{array}$$

where $T=V^{-1}-V^{-1}X{\left(X\prime V^{-1}X\right)}^{-1}X\prime V^{-1}$. These formulas confirm (11) in the main text.

### The proof of Equation (13)

According to Henderson (1975), the best linear unbiased prediction of random effects a and its variance for the model (4) is the following:

(A4) $$\hat{a}=\rho M\prime {\tilde{V}}^{-1}(y-X{\left(X\prime {\tilde{V}}^{-1}X\right)}^{-1}X\prime {\tilde{V}}^{-1}y)$$

(A5) $$\text{Var}(\hat{a})=(\rho I-C_{22})\sigma_{e}^{2},$$

where $\rho =\sigma_{a}^{2}/\sigma_{e}^{2},\;C_{22}$ is the defined as follows:

$${\left(\begin{array}{cc} X\prime X & X\prime M\\ M\prime X & M\prime M+\rho^{-1}I \end{array}\right)}^{-1}=\left(\begin{array}{cc} C_{11} & C_{12}\\ C_{12}^{\prime } & C_{22} \end{array}\right)$$

and $\tilde{V}=I+\rho \mathrm{MM}\prime$.

In fact, $\tilde{V}$ is the same as V defined in the last subsection. Recall the equivalence between (3) and (4), which means that $\sigma_{a}^{2}=\sigma_{g}^{2}$. Hence, we know that $\rho =\sigma_{g}^{2}/\sigma_{e}^{2}=\lambda$. Since G=MM′, we know that $\tilde{V}=I+\lambda G=V$.

Replacing $\tilde{V}$ by V in (A4), we have

(A6) $$\begin{array}{cc} \hat{a} & =\rho M\prime V^{-1}(y-X{\left(X\prime V^{-1}X\right)}^{-1}X\prime V^{-1}y)\\ & =\rho M\prime (V^{-1}-V^{-1}X{\left(X\prime V^{-1}X\right)}^{-1}X\prime V^{-1})y=\frac{\sigma_{a}^{2}}{\sigma_{e}^{2}}M\prime \mathrm{Ty}. \end{array}$$

where $T=V^{-1}-V^{-1}X{\left(X\prime V^{-1}X\right)}^{-1}X\prime V^{-1}$ as in the last subsection.

Using Lemma A.1, we can derive $C_{22}$ as follows:

(A7) $$\begin{array}{cc} C_{22} & ={\left(M\prime M+\rho^{-1}I-M\prime X{\left(X\prime X\right)}^{-1}X\prime M\right)}^{-1}\\ & ={\left(\rho^{-1}I+M\prime (I-X{\left(X\prime X\right)}^{-1}X\prime )M\right)}^{-1}={\left(\frac{\sigma_{e}^{2}}{\sigma_{a}^{2}}I+M\prime \mathrm{SM}\right)}^{-1}, \end{array}$$

where $S=I-X{\left(X\prime X\right)}^{-1}X\prime$.

Thus, replacing $C_{22}$ by (A7) in (A5), we have:

(A8) $$\text{Var}(\hat{a})=\sigma_{a}^{2}I-{\left(\frac{\sigma_{e}^{2}}{\sigma_{a}^{2}}I+M\prime \mathrm{SM}\right)}^{-1}\sigma_{e}^{2}.$$

In view of (A6) and (A8), we have completed the proof.

### The proof of Equation (17)

To achieve our goal, we need to apply the singular value decomposition (SVD) of the matrix X. Assume that the SVD of X is X=UΣW. In the decomposition, U is an *n *×* n* orthogonal matrix, $\Sigma =(D\;0_{k\times (n-k)})\prime$, where D is an *k *×* k* diagonal matrix whose diagonal entries are the singular values of X and $0_{k\times (n-k)}$ is a k×(n−k) matrix of zeros, W is a *k *×* k* orthogonal matrix. We can write $U=(U_{1}\;U_{2})$, where $U_{1}$ is the left *n *×* k* block and $U_{2}$ is the right n×(n−k) block of U. Then we have

(A9) $$X=U\Sigma W=(U_{1}\;U_{2})\left(\begin{array}{c} D\\ 0_{k\times (n-k)} \end{array}\right)W=U_{1}\mathrm{DW}.$$

The orthogonality of U ensures the following:

(A10)
$$\begin{array}{cc} U_{2}^{\prime }U_{1} & =0_{(n-k)\times k},\;\;\;U_{1}^{\prime }U_{2}=0_{k\times (n-k)},\\ U_{1}^{\prime }U_{1} & =I_{k},\;\;\;U_{2}^{\prime }U_{2}=I_{n-k},\;\;\;U_{1}U_{1}^{\prime }+U_{2}U_{2}^{\prime }=I_{n}. \end{array}$$

Using (A9) and (A10), we have

$$\begin{array}{c} S=I-X{\left(X\prime X\right)}^{-1}X\prime =I-U_{1}\mathrm{DW}{\left(W\prime DU_{1}^{\prime }U_{1}\mathrm{DW}\right)}^{-1}W\prime DU_{1}^{\prime }\\ =I-U_{1}\mathrm{DW}(W\prime D^{-2}W)W\prime DU_{1}^{\prime }\\ =I-U_{1}U_{1}^{\prime }=U_{2}U_{2}^{\prime } \end{array}.$$

Replacing S in (A8) by $U_{2}U_{2}^{\prime }$ and using Lemma A.1, we can simplify the formula for $\text{Var}(\hat{a})$ as follows:

$$\begin{array}{c} \text{Var}(\hat{a})=\sigma_{a}^{2}I-{\left(\frac{\sigma_{e}^{2}}{\sigma_{a}^{2}}I+M\prime U_{2}U_{2}^{\prime }M\right)}^{-1}\sigma_{e}^{2}\\ =\sigma_{a}^{2}I-(\rho I-\rho^{2}M\prime U_{2}{\left(I+\rho U_{2}^{\prime }\mathrm{MM}\prime U_{2}\right)}^{-1}U_{2}^{\prime }M)\sigma_{e}^{2}\\ =\frac{\sigma_{a}^{4}}{\sigma_{e}^{2}}M\prime U_{2}{\left(I+\rho U_{2}^{\prime }GU_{2}\right)}^{-1}U_{2}^{\prime }M \end{array}.$$

Using the above formula, we can derive an explicit formula for the *i*-th diagonal element of $\text{Var}(\hat{a})$:

(A11) $$\text{Var}{\left(\hat{a}\right)}_{i,i}=\frac{\sigma_{a}^{4}}{\sigma_{e}^{2}}m_{i}^{\prime }U_{2}{\left(I+\rho U_{2}^{\prime }GU_{2}\right)}^{-1}U_{2}^{\prime }m_{i}.$$

Now, comparing (A11) with (17) and noting that ρ=λ (see the last subsection), we only need to prove:

$$T=U_{2}{\left(I+\lambda U_{2}^{\prime }GU_{2}\right)}^{-1}U_{2}^{\prime }.$$

Using (A9) and (A10), we can calculate that

$$\begin{array}{c} T=V^{-1}-V^{-1}X{\left(X\prime V^{-1}X\right)}^{-1}X\prime V^{-1}\\ =V^{-1}-V^{-1}U_{1}\mathrm{DW}{\left(W\prime DU_{1}^{\prime }V^{-1}U_{1}\mathrm{DW}\right)}^{-1}W\prime DU_{1}^{\prime }V^{-1}\\ =V^{-1}-V^{-1}U_{1}\mathrm{DW}(W\prime D^{-1}{\left(U_{1}^{\prime }V^{-1}U_{1}\right)}^{-1}D^{-1}W)W\prime DU_{1}^{\prime }V^{-1}\\ =V^{-1}-V^{-1}U_{1}{\left(U_{1}^{\prime }V^{-1}U_{1}\right)}^{-1}U_{1}^{\prime }V^{-1}. \end{array}$$

Note that $V^{-1}$ is positive-definite matrix, $U_{1}^{\prime }U_{2}=0$. Thus, we can apply Lemma A.2 to the above formula, yielding:

$$T=U_{2}{\left(U_{2}^{\prime }VU_{2}\right)}^{-1}U_{2}^{\prime }=U_{2}{\left(U_{2}^{\prime }(I+\lambda G)U_{2}\right)}^{-1}U_{2}^{\prime }=U_{2}{\left(I+\lambda U_{2}^{\prime }GU_{2}\right)}^{-1}U_{2}^{\prime },$$

which completes the proof.
